# Ethyl 2-[4,5-bis­(butyl­sulfan­yl)-1,3-dithiol-2-yl­idene]-1,3-dithiolo[4,5-*c*]pyrrole-4-carboxyl­ate

**DOI:** 10.1107/S1600536812015309

**Published:** 2012-04-18

**Authors:** Dong-Feng Li, Xiao-Fei Zhu, Shuang Guan, Rui-Bin Hou

**Affiliations:** a School of Chemistry and Life Science, Changchun University of Technology, Changchun 130012, People’s Republic of China

## Abstract

In the title mol­ecule, C_19_H_25_NO_2_S_6_, the butyl chains are each disordered over two conformations in a 0.689 (10):0.311 (10) ratio. In the crystal, pairs of N—H⋯O hydrogen bonds link mol­ecules into centrosymmetric dimers. Short S⋯S contacts of 3.553 (4) Å are observed.

## Related literature

For background to tetra­thia­fulvalenes, see: Jeppesen *et al.* (1999 ▶); Hansel *et al.* (2004 ▶). For details of the synthesis, see: Hou *et al.* (2010 ▶). For a similar structure, see: Hou & Yin (2010 ▶).
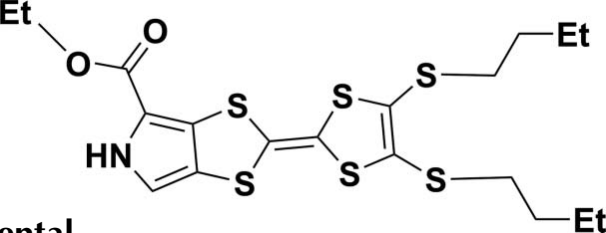


## Experimental

### 

#### Crystal data

C_19_H_25_NO_2_S_6_
*M* *_r_* = 491.76
Monoclinic, 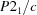

*a* = 9.687 (3) Å
*b* = 26.608 (9) Å
*c* = 8.876 (5) Å
β = 108.408 (17)°
*V* = 2170.7 (15) Å^3^
*Z* = 4
Mo *K*α radiation
μ = 0.65 mm^−1^
*T* = 288 K
0.48 × 0.18 × 0.17 mm

#### Data collection

Rigaku R-AXIS RAPID diffractometer
Absorption correction: multi-scan (*ABSCOR*; Higashi, 1995 ▶) *T*_min_ = 0.747, *T*_max_ = 0.898
17087 measured reflections
3823 independent reflections
3114 reflections with *I* > 2σ(*I*)
*R*_int_ = 0.030

#### Refinement

*R*[*F* ^2^ > 2σ(*F* ^2^)] = 0.042
*wR*(*F* ^2^) = 0.120
*S* = 1.04
3823 reflections
309 parameters
188 restraints
H-atom parameters constrained
Δρ_max_ = 0.39 e Å^−3^
Δρ_min_ = −0.71 e Å^−3^

### 

Data collection: *RAPID-AUTO* (Rigaku, 1998 ▶); cell refinement: *RAPID-AUTO*; data reduction: *CrystalStructure* (Rigaku/MSC, 2002 ▶); program(s) used to solve structure: *SHELXS97* (Sheldrick, 2008 ▶); program(s) used to refine structure: *SHELXL97* (Sheldrick, 2008 ▶); molecular graphics: *SHELXTL* (Sheldrick, 2008 ▶); software used to prepare material for publication: *SHELXL97*.

## Supplementary Material

Crystal structure: contains datablock(s) global, I. DOI: 10.1107/S1600536812015309/cv5262sup1.cif

Structure factors: contains datablock(s) I. DOI: 10.1107/S1600536812015309/cv5262Isup2.hkl

Supplementary material file. DOI: 10.1107/S1600536812015309/cv5262Isup3.cml

Additional supplementary materials:  crystallographic information; 3D view; checkCIF report

## Figures and Tables

**Table 1:** Hydrogen-bond geometry (Å, °)

*D*—H⋯*A*	*D*—H	H⋯*A*	*D*⋯*A*	*D*—H⋯*A*
N1—H1⋯O1^i^	0.86	1.96	2.782 (3)	160

Symmetry code: (i) 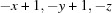.

**Fig. 1.**
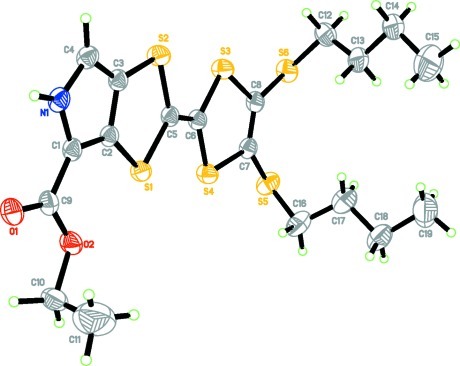
Molecular structure of the title compound with displacement ellipsoids drawn at the 30% probalility level. Only major components of the disordered groups are shown.

## supplementary crystallographic information

### Comment

The tetrathiafulvalenes (TTFs) have attracted considerable attention in organic synthesis (Jeppesen *et al.*, 1999) due to high electrical conductivity and superconductor properties of these compounds. Recently, Hansel reported a series of novel donor-π-acceptor dyads with pyrrolo-annelated tetrathiafulvalene units, which possess significant third-order non-linear optical properties (Hansel *et al.*, 2004). In this paper, we describe the crystal structure of the title compound (I), which is a key precursor of the dyads.

In (I) (Fig. 1), all bond lengths and angles are normal and comparable with the reported ones (Hou & Yin, 2010). Except C11 and two butyls, all non-hydrogen atoms are nearly coplanar. The intermolecular N—H···O hydrogen bonds (Table 1) link the molecuels into dimers, and the dimers are further arranged along [101] direction due to the short S···S [3.553 (4) Å] interactions.

### Experimental

The title compound was prepared according to the literature (Hou *et al.*, 2010). Single crystals suitable for X-ray diffraction were prepared by slow evaporation method from a solution in dichloromethane/petroleum (60–90 °C) at room temperature.

### Refinement

C-bound H-atoms were placed in calculated positions (C—H 0.93-0.97 Å) and were included in the refinement in the riding model, with *U*_iso_(H) = 1.5 or 1.2 *U*_eq_(C). N-bound H-atom was placed in calculated position with N—H = 0.86 Å and refined with *U*_iso_(H) = 1.2 *U*_eq_(N).

### Crystal data

**Table:**

C_19_H_25_NO_2_S_6_	*F*(000) = 1032
*M**_r_* = 491.76	*D*_x_ = 1.505 Mg m^−^^3^
Monoclinic, *P*2_1_/*c*	Mo *K*α radiation, λ = 0.71073 Å
Hall symbol: -P 2ybc	Cell parameters from 15498 reflections
*a* = 9.687 (3) Å	θ = 3.1–27.5°
*b* = 26.608 (9) Å	µ = 0.65 mm^−^^1^
*c* = 8.876 (5) Å	*T* = 288 K
β = 108.408 (17)°	Block, yellow
*V* = 2170.7 (15) Å^3^	0.48 × 0.18 × 0.17 mm
*Z* = 4	

### Data collection

**Table:**

Rigaku R-AXIS RAPID diffractometer	3823 independent reflections
Radiation source: fine-focus sealed tube	3114 reflections with *I* > 2σ(*I*)
Graphite monochromator	*R*_int_ = 0.030
ω scans	θ_max_ = 25.0°, θ_min_ = 3.1°
Absorption correction: multi-scan (*ABSCOR*; Higashi, 1995)	*h* = −11→11
*T*_min_ = 0.747, *T*_max_ = 0.898	*k* = −31→31
17087 measured reflections	*l* = −10→10

### Refinement

**Table:**

Refinement on *F*^2^	Primary atom site location: structure-invariant direct methods
Least-squares matrix: full	Secondary atom site location: difference Fourier map
*R*[*F*^2^ > 2σ(*F*^2^)] = 0.042	Hydrogen site location: inferred from neighbouring sites
*wR*(*F*^2^) = 0.120	H-atom parameters constrained
*S* = 1.04	*w* = 1/[σ^2^(*F*_o_^2^) + (0.0611*P*)^2^ + 1.4202*P*] where *P* = (*F*_o_^2^ + 2*F*_c_^2^)/3
3823 reflections	(Δ/σ)_max_ = 0.001
309 parameters	Δρ_max_ = 0.39 e Å^−^^3^
188 restraints	Δρ_min_ = −0.71 e Å^−^^3^

### Special details

**Table:**

Experimental. (See detailed section in the paper)
Geometry. All e.s.d.'s (except the e.s.d. in the dihedral angle between two l.s. planes) are estimated using the full covariance matrix. The cell e.s.d.'s are taken into account individually in the estimation of e.s.d.'s in distances, angles and torsion angles; correlations between e.s.d.'s in cell parameters are only used when they are defined by crystal symmetry. An approximate (isotropic) treatment of cell e.s.d.'s is used for estimating e.s.d.'s involving l.s. planes.
Refinement. Refinement of *F*^2^ against ALL reflections. The weighted *R*-factor *wR* and goodness of fit *S* are based on *F*^2^, conventional *R*-factors *R* are based on *F*, with *F* set to zero for negative *F*^2^. The threshold expression of *F*^2^ > σ(*F*^2^) is used only for calculating *R*-factors(gt) *etc*. and is not relevant to the choice of reflections for refinement. *R*-factors based on *F*^2^ are statistically about twice as large as those based on *F*, and *R*- factors based on ALL data will be even larger.

### Fractional atomic coordinates and isotropic or equivalent isotropic displacement parameters (Å^2^)

**Table:**

	*x*	*y*	*z*	*U*_iso_*/*U*_eq_	Occ. (<1)
C1	0.4627 (3)	0.46393 (10)	0.2578 (3)	0.0442 (6)	
C2	0.3963 (3)	0.45459 (9)	0.3722 (3)	0.0413 (6)	
C3	0.2598 (3)	0.47643 (10)	0.3237 (3)	0.0448 (6)	
C4	0.2437 (3)	0.49832 (11)	0.1810 (3)	0.0486 (7)	
H4	0.1619	0.5157	0.1197	0.058*	
C5	0.2830 (3)	0.43726 (10)	0.5907 (3)	0.0437 (6)	
C6	0.2589 (3)	0.42284 (10)	0.7241 (3)	0.0460 (6)	
C7	0.2628 (4)	0.37832 (11)	0.9810 (3)	0.0491 (7)	
C8	0.1321 (3)	0.39944 (11)	0.9317 (3)	0.0491 (7)	
C9	0.6006 (3)	0.44958 (11)	0.2450 (3)	0.0474 (7)	
C10	0.8040 (4)	0.40090 (14)	0.3572 (5)	0.0669 (9)	
H10A	0.8668	0.3919	0.4624	0.080*	
H10B	0.8542	0.4251	0.3114	0.080*	
C11	0.7626 (7)	0.3553 (2)	0.2540 (8)	0.151 (3)	
H11A	0.7100	0.3324	0.2992	0.227*	
H11B	0.8489	0.3392	0.2466	0.227*	
H11C	0.7024	0.3651	0.1499	0.227*	
S6	0.00077 (10)	0.39799 (4)	1.02888 (11)	0.0656 (3)	
C12	−0.1593 (4)	0.37886 (14)	0.8701 (5)	0.0757 (10)	
H12A	−0.2410	0.3777	0.9110	0.091*	0.689 (10)
H12B	−0.1806	0.4043	0.7876	0.091*	0.689 (10)
H12C	−0.2459	0.3855	0.8995	0.091*	0.311 (10)
H12D	−0.1662	0.3980	0.7749	0.091*	0.311 (10)
C13	−0.1481 (11)	0.3310 (2)	0.7998 (12)	0.079 (2)	0.689 (10)
H13A	−0.1252	0.3055	0.8820	0.095*	0.689 (10)
H13B	−0.0684	0.3322	0.7557	0.095*	0.689 (10)
C14	−0.2837 (10)	0.3163 (3)	0.6721 (11)	0.084 (2)	0.689 (10)
H14A	−0.3045	0.3393	0.5830	0.101*	0.689 (10)
H14B	−0.3662	0.3155	0.7117	0.101*	0.689 (10)
C15	−0.249 (2)	0.2639 (4)	0.625 (2)	0.179 (8)	0.689 (10)
H15A	−0.3317	0.2511	0.5421	0.268*	0.689 (10)
H15B	−0.2273	0.2420	0.7150	0.268*	0.689 (10)
H15C	−0.1668	0.2656	0.5866	0.268*	0.689 (10)
C13'	−0.145 (3)	0.3255 (4)	0.850 (4)	0.122 (10)	0.311 (10)
H13C	−0.1223	0.3089	0.9524	0.147*	0.311 (10)
H13D	−0.0655	0.3192	0.8079	0.147*	0.311 (10)
C14'	−0.282 (3)	0.3044 (10)	0.740 (3)	0.138 (8)	0.311 (10)
H14C	−0.3494	0.3320	0.7049	0.165*	0.311 (10)
H14D	−0.3225	0.2822	0.8020	0.165*	0.311 (10)
C15'	−0.279 (3)	0.2759 (10)	0.595 (3)	0.105 (6)	0.311 (10)
H15D	−0.3770	0.2674	0.5330	0.157*	0.311 (10)
H15E	−0.2233	0.2457	0.6266	0.157*	0.311 (10)
H15F	−0.2361	0.2964	0.5331	0.157*	0.311 (10)
S5	0.33144 (12)	0.34689 (4)	1.15800 (11)	0.0716 (3)	
C16	0.3736 (6)	0.29134 (14)	1.0928 (6)	0.0958 (14)	
H16A	0.4135	0.2961	1.0065	0.115*	0.689 (10)
H16B	0.4436	0.2731	1.1779	0.115*	0.689 (10)
H16C	0.4545	0.2981	1.0536	0.115*	0.311 (10)
H16D	0.4117	0.2703	1.1861	0.115*	0.311 (10)
C17	0.2338 (8)	0.2642 (2)	1.0379 (10)	0.089 (2)	0.689 (10)
H17A	0.1821	0.2680	1.1147	0.106*	0.689 (10)
H17B	0.1737	0.2780	0.9374	0.106*	0.689 (10)
C18	0.2631 (8)	0.2107 (2)	1.0189 (10)	0.092 (2)	0.689 (10)
H18A	0.3163	0.1958	1.1204	0.111*	0.689 (10)
H18B	0.3194	0.2066	0.9467	0.111*	0.689 (10)
C19	0.1163 (15)	0.1872 (6)	0.952 (3)	0.132 (6)	0.689 (10)
H19A	0.1271	0.1521	0.9337	0.198*	0.689 (10)
H19B	0.0641	0.2033	0.8542	0.198*	0.689 (10)
H19C	0.0632	0.1910	1.0266	0.198*	0.689 (10)
C17'	0.2867 (19)	0.2577 (5)	0.9740 (15)	0.105 (5)	0.311 (10)
H17C	0.3450	0.2356	0.9319	0.126*	0.311 (10)
H17D	0.2157	0.2752	0.8880	0.126*	0.311 (10)
C18'	0.219 (3)	0.2313 (8)	1.081 (2)	0.127 (6)	0.311 (10)
H18C	0.1472	0.2527	1.1031	0.152*	0.311 (10)
H18D	0.2931	0.2231	1.1803	0.152*	0.311 (10)
C19'	0.149 (3)	0.1841 (9)	0.999 (5)	0.118 (10)	0.311 (10)
H19D	0.1170	0.1641	1.0714	0.176*	0.311 (10)
H19E	0.2182	0.1654	0.9641	0.176*	0.311 (10)
H19F	0.0671	0.1927	0.9087	0.176*	0.311 (10)
N1	0.3666 (3)	0.49065 (8)	0.1432 (3)	0.0486 (6)	
H1	0.3813	0.5013	0.0580	0.058*	
O1	0.6480 (2)	0.46096 (8)	0.1393 (3)	0.0581 (6)	
O2	0.6701 (2)	0.42077 (8)	0.3638 (2)	0.0540 (5)	
S1	0.44602 (8)	0.42390 (3)	0.54986 (9)	0.0483 (2)	
S2	0.15379 (9)	0.47146 (3)	0.44873 (10)	0.0558 (2)	
S3	0.09887 (9)	0.43714 (3)	0.76775 (9)	0.0564 (2)	
S4	0.38360 (9)	0.39046 (3)	0.87406 (10)	0.0548 (2)	

### Atomic displacement parameters (Å^2^)

**Table:**

	*U*^11^	*U*^22^	*U*^33^	*U*^12^	*U*^13^	*U*^23^
C1	0.0517 (17)	0.0411 (14)	0.0481 (15)	−0.0027 (12)	0.0276 (13)	0.0018 (12)
C2	0.0472 (16)	0.0381 (13)	0.0444 (14)	−0.0021 (11)	0.0229 (13)	0.0003 (11)
C3	0.0484 (17)	0.0425 (14)	0.0506 (16)	−0.0013 (12)	0.0255 (13)	0.0013 (12)
C4	0.0568 (18)	0.0460 (15)	0.0491 (16)	0.0038 (13)	0.0251 (14)	0.0055 (12)
C5	0.0471 (16)	0.0429 (14)	0.0487 (15)	−0.0026 (12)	0.0259 (13)	0.0010 (12)
C6	0.0496 (17)	0.0474 (15)	0.0467 (16)	−0.0024 (12)	0.0235 (13)	0.0010 (12)
C7	0.0605 (19)	0.0465 (15)	0.0455 (16)	−0.0039 (13)	0.0244 (14)	0.0016 (12)
C8	0.0568 (19)	0.0524 (16)	0.0460 (16)	−0.0051 (14)	0.0277 (14)	0.0034 (13)
C9	0.0504 (17)	0.0474 (15)	0.0500 (16)	−0.0085 (13)	0.0238 (14)	−0.0008 (13)
C10	0.0492 (19)	0.082 (2)	0.075 (2)	0.0094 (17)	0.0266 (17)	0.0141 (19)
C11	0.134 (6)	0.165 (6)	0.179 (7)	0.040 (5)	0.084 (5)	0.022 (5)
S6	0.0676 (6)	0.0819 (6)	0.0619 (5)	−0.0029 (4)	0.0415 (5)	0.0085 (4)
C12	0.063 (2)	0.080 (2)	0.096 (3)	−0.0058 (18)	0.042 (2)	0.006 (2)
C13	0.075 (4)	0.067 (3)	0.104 (6)	−0.010 (3)	0.041 (4)	0.014 (3)
C14	0.072 (4)	0.072 (4)	0.113 (6)	−0.011 (3)	0.037 (4)	0.010 (4)
C15	0.164 (15)	0.094 (8)	0.266 (18)	−0.016 (8)	0.050 (13)	−0.060 (10)
C13'	0.115 (14)	0.089 (9)	0.149 (17)	0.001 (10)	0.021 (13)	0.001 (11)
C14'	0.133 (14)	0.094 (12)	0.166 (17)	−0.024 (11)	0.018 (14)	−0.004 (12)
C15'	0.081 (12)	0.099 (15)	0.132 (12)	−0.022 (11)	0.029 (11)	0.030 (10)
S5	0.0952 (7)	0.0666 (5)	0.0576 (5)	0.0101 (5)	0.0307 (5)	0.0186 (4)
C16	0.120 (4)	0.060 (2)	0.105 (3)	0.011 (2)	0.031 (3)	0.018 (2)
C17	0.112 (5)	0.067 (4)	0.090 (5)	0.007 (3)	0.037 (4)	−0.004 (3)
C18	0.121 (5)	0.060 (3)	0.107 (5)	0.007 (4)	0.051 (4)	−0.003 (3)
C19	0.133 (10)	0.103 (9)	0.152 (11)	−0.035 (9)	0.035 (9)	−0.005 (8)
C17'	0.152 (12)	0.076 (9)	0.122 (10)	−0.009 (7)	0.092 (9)	−0.034 (7)
C18'	0.173 (15)	0.102 (12)	0.140 (13)	−0.015 (10)	0.098 (11)	−0.019 (10)
C19'	0.110 (16)	0.043 (8)	0.20 (3)	0.021 (7)	0.046 (16)	0.032 (10)
N1	0.0611 (16)	0.0467 (13)	0.0473 (13)	0.0003 (11)	0.0302 (12)	0.0063 (11)
O1	0.0600 (13)	0.0686 (13)	0.0588 (13)	−0.0016 (11)	0.0373 (11)	0.0114 (10)
O2	0.0493 (12)	0.0639 (13)	0.0566 (12)	0.0012 (10)	0.0281 (10)	0.0102 (10)
S1	0.0495 (4)	0.0527 (4)	0.0506 (4)	0.0049 (3)	0.0271 (3)	0.0094 (3)
S2	0.0520 (5)	0.0668 (5)	0.0599 (5)	0.0112 (4)	0.0336 (4)	0.0159 (4)
S3	0.0546 (5)	0.0706 (5)	0.0541 (5)	0.0094 (4)	0.0315 (4)	0.0154 (4)
S4	0.0523 (5)	0.0657 (5)	0.0522 (4)	0.0051 (4)	0.0247 (4)	0.0095 (4)

### Geometric parameters (Å, º)

**Table:**

C1—N1	1.345 (4)	C14—H14B	0.9700
C1—C2	1.385 (4)	C15—H15A	0.9600
C1—C9	1.427 (4)	C15—H15B	0.9600
C2—C3	1.383 (4)	C15—H15C	0.9600
C2—S1	1.704 (3)	C13'—C14'	1.484 (9)
C3—C4	1.358 (4)	C13'—H13C	0.9700
C3—S2	1.738 (3)	C13'—H13D	0.9700
C4—N1	1.350 (4)	C14'—C15'	1.501 (9)
C4—H4	0.9300	C14'—H14C	0.9700
C5—C6	1.333 (4)	C14'—H14D	0.9700
C5—S2	1.728 (3)	C15'—H15D	0.9600
C5—S1	1.765 (3)	C15'—H15E	0.9600
C6—S4	1.720 (3)	C15'—H15F	0.9600
C6—S3	1.754 (3)	S5—C16	1.684 (4)
C7—C8	1.327 (4)	C16—C17'	1.435 (9)
C7—S5	1.718 (3)	C16—C17	1.475 (7)
C7—S4	1.753 (3)	C16—H16A	0.9700
C8—S3	1.713 (3)	C16—H16B	0.9700
C8—S6	1.748 (3)	C16—H16C	0.9700
C9—O1	1.206 (3)	C16—H16D	0.9700
C9—O2	1.306 (4)	C17—C18	1.473 (7)
C10—O2	1.419 (4)	C17—H17A	0.9700
C10—C11	1.496 (4)	C17—H17B	0.9700
C10—H10A	0.9700	C18—C19	1.495 (8)
C10—H10B	0.9700	C18—H18A	0.9700
C11—H11A	0.9600	C18—H18B	0.9700
C11—H11B	0.9600	C19—H19A	0.9600
C11—H11C	0.9600	C19—H19B	0.9600
S6—C12	1.808 (4)	C19—H19C	0.9600
C12—C13	1.437 (6)	C17'—C18'	1.485 (9)
C12—C13'	1.444 (9)	C17'—H17C	0.9700
C12—H12A	0.9700	C17'—H17D	0.9700
C12—H12B	0.9700	C18'—C19'	1.503 (9)
C12—H12C	0.9700	C18'—H18C	0.9700
C12—H12D	0.9700	C18'—H18D	0.9700
C13—C14	1.491 (7)	C19'—H19D	0.9600
C13—H13A	0.9700	C19'—H19E	0.9600
C13—H13B	0.9700	C19'—H19F	0.9600
C14—C15	1.524 (4)	N1—H1	0.8600
C14—H14A	0.9700		
			
N1—C1—C2	106.2 (2)	C12—C13'—H13D	109.5
N1—C1—C9	121.5 (2)	C14'—C13'—H13D	109.5
C2—C1—C9	132.3 (3)	H13C—C13'—H13D	108.1
C3—C2—C1	108.4 (3)	C13'—C14'—C15'	120 (3)
C3—C2—S1	116.9 (2)	C13'—C14'—H14C	107.3
C1—C2—S1	134.7 (2)	C15'—C14'—H14C	107.3
C4—C3—C2	106.7 (3)	C13'—C14'—H14D	107.3
C4—C3—S2	135.3 (2)	C15'—C14'—H14D	107.3
C2—C3—S2	118.0 (2)	H14C—C14'—H14D	106.9
N1—C4—C3	108.5 (3)	C14'—C15'—H15D	109.5
N1—C4—H4	125.8	C14'—C15'—H15E	109.5
C3—C4—H4	125.8	H15D—C15'—H15E	109.5
C6—C5—S2	120.6 (2)	C14'—C15'—H15F	109.5
C6—C5—S1	122.9 (2)	H15D—C15'—H15F	109.5
S2—C5—S1	116.44 (15)	H15E—C15'—H15F	109.5
C5—C6—S4	123.6 (2)	C16—S5—C7	100.3 (2)
C5—C6—S3	123.3 (2)	C17'—C16—C17	35.7 (6)
S4—C6—S3	113.05 (15)	C17'—C16—S5	130.5 (8)
C8—C7—S5	124.1 (2)	C17—C16—S5	104.2 (4)
C8—C7—S4	118.3 (2)	C17'—C16—H16A	77.8
S5—C7—S4	116.94 (19)	C17—C16—H16A	110.9
C7—C8—S3	115.6 (2)	S5—C16—H16A	110.9
C7—C8—S6	126.6 (2)	C17'—C16—H16B	111.4
S3—C8—S6	117.47 (18)	C17—C16—H16B	110.9
O1—C9—O2	123.3 (3)	S5—C16—H16B	110.9
O1—C9—C1	126.2 (3)	H16A—C16—H16B	108.9
O2—C9—C1	110.5 (2)	C17'—C16—H16C	101.8
O2—C10—C11	104.8 (3)	C17—C16—H16C	137.3
O2—C10—H10A	110.8	S5—C16—H16C	105.7
C11—C10—H10A	110.8	H16A—C16—H16C	28.6
O2—C10—H10B	110.8	H16B—C16—H16C	85.8
C11—C10—H10B	110.8	C17'—C16—H16D	104.2
H10A—C10—H10B	108.9	C17—C16—H16D	93.6
C10—C11—H11A	109.5	S5—C16—H16D	106.3
C10—C11—H11B	109.5	H16A—C16—H16D	127.6
H11A—C11—H11B	109.5	H16B—C16—H16D	20.6
C10—C11—H11C	109.5	H16C—C16—H16D	106.3
H11A—C11—H11C	109.5	C18—C17—C16	108.7 (6)
H11B—C11—H11C	109.5	C18—C17—H17A	110.0
C8—S6—C12	101.55 (17)	C16—C17—H17A	110.0
C13—C12—C13'	18.5 (16)	C18—C17—H17B	110.0
C13—C12—S6	115.1 (5)	C16—C17—H17B	110.0
C13'—C12—S6	106.3 (12)	H17A—C17—H17B	108.3
C13—C12—H12A	108.5	C17—C18—C19	104.9 (9)
C13'—C12—H12A	97.8	C17—C18—H18A	110.8
S6—C12—H12A	108.5	C19—C18—H18A	110.8
C13—C12—H12B	108.5	C17—C18—H18B	110.8
C13'—C12—H12B	126.8	C19—C18—H18B	110.8
S6—C12—H12B	108.5	H18A—C18—H18B	108.9
H12A—C12—H12B	107.5	C18—C19—H19A	109.5
C13—C12—H12C	117.9	C18—C19—H19B	109.5
C13'—C12—H12C	109.6	H19A—C19—H19B	109.5
S6—C12—H12C	109.9	C18—C19—H19C	109.5
H12A—C12—H12C	13.5	H19A—C19—H19C	109.5
H12B—C12—H12C	94.7	H19B—C19—H19C	109.5
C13—C12—H12D	94.6	C16—C17'—C18'	95.7 (11)
C13'—C12—H12D	113.1	C16—C17'—H17C	112.6
S6—C12—H12D	109.7	C18'—C17'—H17C	112.6
H12A—C12—H12D	120.2	C16—C17'—H17D	112.6
H12B—C12—H12D	15.7	C18'—C17'—H17D	112.6
H12C—C12—H12D	108.2	H17C—C17'—H17D	110.1
C12—C13—C14	113.2 (7)	C17'—C18'—C19'	108 (2)
C12—C13—H13A	108.9	C17'—C18'—H18C	110.1
C14—C13—H13A	108.9	C19'—C18'—H18C	110.1
C12—C13—H13B	108.9	C17'—C18'—H18D	110.1
C14—C13—H13B	108.9	C19'—C18'—H18D	110.1
H13A—C13—H13B	107.8	H18C—C18'—H18D	108.4
C13—C14—C15	103.5 (10)	C18'—C19'—H19D	109.5
C13—C14—H14A	111.1	C18'—C19'—H19E	109.5
C15—C14—H14A	111.1	H19D—C19'—H19E	109.5
C13—C14—H14B	111.1	C18'—C19'—H19F	109.5
C15—C14—H14B	111.1	H19D—C19'—H19F	109.5
H14A—C14—H14B	109.0	H19E—C19'—H19F	109.5
C14—C15—H15A	109.5	C1—N1—C4	110.2 (2)
C14—C15—H15B	109.5	C1—N1—H1	124.9
H15A—C15—H15B	109.5	C4—N1—H1	124.9
C14—C15—H15C	109.5	C9—O2—C10	115.5 (2)
H15A—C15—H15C	109.5	C2—S1—C5	94.82 (13)
H15B—C15—H15C	109.5	C5—S2—C3	93.89 (14)
C12—C13'—C14'	110.6 (18)	C8—S3—C6	96.81 (14)
C12—C13'—H13C	109.5	C6—S4—C7	94.90 (14)
C14'—C13'—H13C	109.5		

### Hydrogen-bond geometry (Å, º)

**Table:**

*D*—H···*A*	*D*—H	H···*A*	*D*···*A*	*D*—H···*A*
N1—H1···O1^i^	0.86	1.96	2.782 (3)	160

Symmetry code: (i) −*x*+1, −*y*+1, −*z*.
